# Successful Therapeutic Leukapheresis for Chronic Myeloid Leukemia Identified by Persistent Erection: A Case Report

**DOI:** 10.7759/cureus.61351

**Published:** 2024-05-30

**Authors:** Ryo Kamidani, Naokazu Chiba, Ayumi Kuroda, Akihiro Uchida, Hideshi Okada

**Affiliations:** 1 Department of Emergency and Disaster Medicine, Gifu University, Gifu, JPN

**Keywords:** chronic myeloid leukemia, spectra optia®︎, persistent erection, leukostasis, leukapheresis

## Abstract

Cytoreduction in leukostasis can be achieved using leukapheresis. We report a case of chronic myeloid leukemia (CML) identified by a persistent erection, which was successfully treated using the Spectra Optia®︎ apheresis system. A 29-year-old male presented with an erection for 12 hours without identified triggers and no improvement despite penile corpus cavernosum puncture. His white blood cell count was 458,930/μL. A diagnosis of CML-induced persistent erection with secondary hyperleukocytosis was established. Following an emergency bilateral penile corpus cavernosum incision (distal shunting), he received hydroxyurea and febuxostat. Persistent erection resolved after leukapheresis for two consecutive days. Rapid leukocyte count reduction can effectively address leukostasis in CML without major complications.

## Introduction

Priapism is a rare urological emergency disease characterized by a prolonged and persistent painful erection that does not lead to ejaculation [1]. It is classified as ischemic (low flow) or non-ischemic (high flow). Leukostasis due to hematologic tumors can cause ischemic priapism. Aspiration or shunt surgery for leukostasis warrants supplementation with chemotherapy and leukapheresis to address its underlying cause.

Leukapheresis is performed for the cytoreduction of leukostasis, which is also called symptomatic hyperleukocytosis. Several types of leukemia cause hyperleukocytosis, whereas leukostasis presents decreased tissue perfusion, disseminated intravascular coagulation, intracranial hemorrhage, and respiratory or neurological distress [2]. Leukapheresis is combined with febuxostat or other agents to reduce the risk of tumor lysis syndrome (TLS) and initiate chemotherapy without delay.

Herein, we present a case of chronic myeloid leukemia (CML) identified by a persistent erection successfully treated with the Spectra Optia Apheresis System®︎ (Terumo BCT, Lakewood, United States) in combination with emergency bilateral penile corpus cavernosum incision (distal shunting) and hydroxyurea [3].

## Case presentation

A 29-year-old male without prior comorbidities complained of muscle pain in his extremities for one month, general malaise and fatigue for one week, and a painless persistent erection for 12 hours without triggers, such as trauma, infection, drug use, or persistent sexual arousal. The patient initially underwent penile corpus cavernosum puncture without improvement. He was referred to our hospital with a diagnosis of ischemic persistent erection. His medical history included the excision of a benign tumor in the left auditory nerve, with no other leukemia-related symptoms.

The following physical examination parameters were recorded upon admission: heart rate: 91 bpm; blood pressure: 124/68 mmHg; respiratory rate: 18 breaths/min; SpO_2_: 97% on room air; and body temperature: 36.9℃. His level of consciousness was clear. The spleen was enlarged, 11 cm below the costal margin, and soft in consistency. Hematological parameters were as follows. white blood cell (WBC) counts were markedly increased, and peripheral blood smears showed immature blasts. Lactate dehydrogenase, D-dimer, soluble interleukin-2 receptor, and C-reactive protein were elevated. Hemoglobin was normal. The remaining parameters were within normal ranges (Table 1).

**Table 1:** Laboratory Findings at the Time of Admission

Parameter	Value	Reference range
< Biochemistry >		
Total Protein	8.0 g/dL	6.7-8.3 g/dL
Albumin	4.5 g/dL	3.8-5.2 g/dL
Creatinine kinase	25 IU/L	56-244 IU/L
Aspartate transaminase	33 IU/L	10-40 IU/L
Alanine transaminase	31 IU/L	5-40 IU/L
Lactate dehydrogenase	1,234 IU/L	115-245 IU/L
Alkaline phosphatase	626 IU/L	115-359 IU/L
Γ-glutamyl transpeptidase	149 IU/L	16-73 IU/L
Amylase	23 IU/L	42-114 IU/L
Uric acid	547 μmol/L	0-416 μmol/L
Total bilirubin	22.2 mg/dL	5.1-20.5 μmol/L
Creatinine	69.8 μmol/L	41.5-69.8 μmol/L
Blood urea nitrogen	3.5 mmol/L	2.8-7.8 mmol/L
Sodium	141 mmol/L	136-147 mmol/L
Potassium	3.3 mmol/L	3.6-5.0 mmol/L
Chloride	104 mmol/L	98-109 mmol/L
Inorganic phosphorus	1.3 mmol/L	0.8-1.5 mmol/L
Calcium	2.5 mmol/L	2.2-2.5 mmol/L
Glucose	6.8 mmol/L	3.8-6.0 mmol/L
Hemoglobin A1c	36.6 mmol/mol	26.7-44.2 mmol/mol
C-reactive protein	21,000 μg/L	≦3,000 μg/L
Soluble interleukin-2 receptor	1,706 IU/mL	157-474 IU/mL
< Complete Blood Count >		
White Blood Cells	459×10^9^ cells/L	3.5-9.1×10^9^ cells/L
Neutrophils	45.5%	36.0-69.0%
Lymphocytes	2.0%	27.0-53.0%
Basophils	3.0%	0.0-2.0%
Metamyelocytes	6.5%	0.0%
Myelocytes	35%	0.0%
Promyelocytes	4.5%	0.0%
Blasts	3.0%	0.0%
Red Blood Cells	3.3×10^12^ cells/L	3.7-5.0×10^12^ cells/L
Hemoglobin	98 g/L	113-152 g/L
Hematocrit	0.3 /L	0.4-0.5 /L
Platelet	249×10^9^ cells/L	130-369×10^9^ cells/L
< Coagulation Status >		
Activated partial thromboplastin time	27.6 sec	24.3-36.0 sec
Prothrombin time-international normalized ratio	1.05	0.85-1.15
D-dimer	291 µg/L	<1,000 µg/L
< Blood Gas of Penile Corpus Cavernosum >		
pH	6.497	7.35-7.45
PaCO_2_	148 mmHg	32-45 mmHg
PaO_2_	7.5 mmHg	
HCO_3_^-^	10.8 mmol/L	20-26 mmol/L
Base Excess	-21.4 mmol/L	-3.3-+1.2 mmol/L

**Figure 1:**
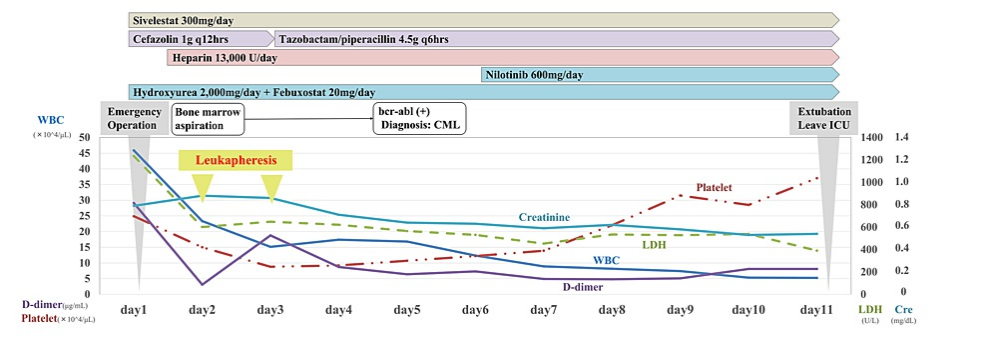
Clinical Course WBC: white blood cell; LDH: lactic acid dehydrogenase; Cre: creatinine; ICU: intensive care unit; BCR-ABL: breakpoint cluster region-Abelson proto-oncogene; CML: chronic myeloid leukemia

The patient was determined to have persistent erections due to secondary hyperleukocytosis and was provisionally diagnosed with CML as the cause. Urologists performed emergency surgery on the day of admission (day 1). After confirming the venous origin of puncture blood from the penile root, a distal shunting was performed, in which an incision was made from the glans head along the penile corpuscles of the two legs to debridement, followed by visualization of arterial blood flow and suturing the glans head skin incision wound. Upon intensive care unit admission, the patient remained sedated (Figure 1) and was administered hydroxyurea (2,000 mg), febuxostat (20 mg), continuous unfractionated heparin (13,000 units), and sivelestat (300 mg) daily.

On days 2 and 3, leukapheresis was performed with a double-lumen catheter placed in the right femoral vein using the Spectra Optia® system. Acid-citrate-dextrose (15:1) was employed as the anticoagulant at an inlet flow rate of 50 mL/min, with isotonic albumin used as replacement fluid. The first session comprised 2.4 total blood volume (TBV), and the WBC count decreased by 81,960/μL (35.1%); the second session comprised 3.0 TBV, with a WBC increment of 11,070/μL (-7.3%). The persistent erection was resolved on day 2. The bone marrow examination performed on the same day revealed hypercellular status and immature myeloid leukocytosis. On day 5, bone marrow cytogenetics showed the Philadelphia chromosome in 100% of metaphases and the real-time polymerase chain reaction study on peripheral blood for breakpoint cluster region/Abelson proto-oncogene (BCR-ABL) transcript was positive, confirming the CML diagnosis. The various prognostic scores for CML patients were as follows. Sokal score [4] was 0.95 (intermediate risk), the Hasford score [5] was 861.8 (intermediate risk), the European Treatment and Outcome Study (EUTOS) score [6] was 65 (low risk), and the EUTOS long-term survival (ELTS) score [7] was 1.88 (intermediate risk). Nilotinib (600 mg daily) therapy was initiated on day 6. The patient’s condition gradually improved, and he was discharged on day 11. In subsequent outpatient follow-up, he had no complaints of erectile dysfunction.

## Discussion

Ischemic priapism may result from embolic venous obstruction, increased blood hyperviscosity, or venous obstruction of the corpora cavernosa due to an enlarged spleen [8]. Herein, given the absence of splenomegaly and positive BCR-ABL fusion gene, priapism could be attributed to venous obstruction or poor venous outflow due to CML-induced hyperviscosity. Priapism affects <3% of patients with CML, with 27.4% developing permanent erectile dysfunction, and this number rises to 90% if the condition persists over 24 hours [8]. Hence, prompt, structured interventions are essential. The American Urological Association strongly recommends focal therapy of ischemic priapism with intra-cavernosal aspiration or phenylephrine irrigation as the first step [9]. However, the longer the duration, the worse the response to phenylephrine irrigation. If these fail, distal and even proximal shunting are recommended as options. Other options include bilateral pudendal artery ligation, bilateral pudendal artery embolization, chemotherapy, and leukapheresis. As urologic treatment alone fails to relieve hyperleukocytosis, the underlying cause of priapism, a combined approach with chemotherapy is frequently selected. Conversely, improvement with oncological agents alone has been reported, although symptom resolution is delayed [10]. Herein, prompt bilateral penile corpus cavernosum incision (distal shunting) was performed because aspiration was ineffective and erection duration was long; we combined leukapheresis with pharmacotherapy for rapid and safe cytoreduction to preserve sexual function.

Hyperleukocytosis, WBCs >100,000/mL caused by leukemic cell proliferation, often increases morbidity and mortality. Most hyperleukocytosis cases are associated with acute myeloid leukemia, although CML-related cases have been reported [11,12]. Leukostasis describes the pathogenesis of symptomatic hyperleukocytosis, a syndrome characterized by multiple organ failure, coagulopathy, and TLS. To prevent leukostasis, leukapheresis or mechanical WBC removal is undertaken in patients with acute leukemia or hyperleukocytosis-related complications. Mechanical granulocyte and monocyte removal can reduce inflammatory cytokine production and induce a long-term disease course by avoiding drug therapy. Spectra Optia®︎ separates WBCs and their precursors from the blood while returning plasma, platelets, and red blood cells to the patient [11]. The safety and efficacy of Spectra Optia®︎ for leukocyte removal were established in a multicenter study, including 21.4% of patients with CML [13]. Noushad et al. reported a case of successful leukocyte removal using Spectra Optia®︎ to improve penile pain caused by CML-induced priapism that did not resolve with aspiration and phenylephrine irrigation [1].

In hyperleukocytosis, leukapheresis does not substantially impact the early mortality rate, and its efficacy remains uncertain. Although indications and initiation timing remain controversial, leukapheresis is typically initiated when the blast count exceeds 100,000/MMC, leukostasis symptoms are detected, and WBCs decrease by 20-50%. Effective leukapheresis typically requires an apheresis volume exceeding 1.5-2 TBV, and the apheresis volume was 2.4-3 TBV in this case. The WBC count increased after the second apheresis, and chemotherapy was ineffective. The lack of accompanying blast cell destruction in the bone marrow suggests a rebound increase in WBCs following mobilization to the peripheral blood during bone marrow puncture and critical care management. No marked repopulation occurred, suggesting the desired effect was achieved.

## Conclusions

To the best of our knowledge, this is the first report combining aspiration, bilateral penile cavernotomy (distal shunting), chemotherapy, and leukapheresis using Spectra Optia®︎, that suppressed a persistent erection, not pain. Local urological therapy alone or leukapheresis alone would not be the first choice. Immediate WBC reduction and rapid improvement of persistent erection without major complications, such as TLS or disseminated intravascular coagulation, warrant local urological therapy with multidisciplinary treatment, combining leukapheresis, chemotherapy, and radiation therapy.
